# CK14 Expression Identifies a Basal/Squamous-Like Type of Papillary Non-Muscle-Invasive Upper Tract Urothelial Carcinoma

**DOI:** 10.3389/fonc.2020.00623

**Published:** 2020-04-24

**Authors:** Minsun Jung, Insoon Jang, Kwangsoo Kim, Kyung Chul Moon

**Affiliations:** ^1^Department of Pathology, Seoul National University Hospital, Seoul, South Korea; ^2^Division of Clinical Bioinformatics, Biomedical Research Institute, Seoul National University Hospital, Seoul, South Korea; ^3^Medical Research Center, Kidney Research Institute, Seoul National University College of Medicine, Seoul, South Korea

**Keywords:** Cytokeratin 14, carcinoma, transitional cell, upper tract urothelial carcinoma, prognosis, gene expression profiling, high-throughput nucleotide sequencing, basal/squamous-like

## Abstract

**Object:** CK14 expression is an important marker of basal/squamous-like (BASQ)-type muscle-invasive bladder carcinoma, and this molecularly defined subtype has a poor prognosis and a distinct response to chemotherapy. However, CK14 expression and its clinicopathological and molecular significance in papillary non-muscle-invasive upper tract urothelial carcinoma (NMIUTUC) remain unknown. Herein, we investigated the prognostic implications of immunohistochemical (IHC) staining for CK14 and the transcriptional characteristics associated with CK14 expression in papillary NMIUTUC.

**Materials and Methods:** IHC staining for CK14 was conducted in 204 papillary NMIUTUC specimens. Positive CK14 IHC staining was defined as a positive signal in >0% of tumor cells. RNA sequencing data were analyzed from 8 papillary high-grade NMIUTUC specimens consisting of 4 CK14-positive and 4 CK14-negative tumors.

**Results:** CK14 positivity was associated with a high TNM stage (*p* < 0.001) and a high World Health Organization grade (*p* = 0.003). Survival analysis showed that CK14 positivity was significantly associated with poor progression-free survival (*p* = 0.015; hazard ratio [HR] = 2.990; 95% confidence interval [CI] = 1.180–7.580) and was marginally associated with poor cancer-specific survival (*p* = 0.052; HR = 3.77; 95% CI = 0.900–15.780). Gene set enrichment analysis demonstrated that the CK14-positive tumors were associated with a basal subtype of breast cancer, squamous cell carcinoma development, p40, tumor necrosis factor α-nuclear factor-κB, and p53 pathways, and embryonic stem cells; these characteristics are reminiscent of the BASQ subtype. In addition, with a *p* < 0.05 and |fold change| ≥2 as the cutoffs, we identified 178 differentially expressed genes when comparing CK14-positive and CK14-negative tumors. Functional analysis of these genes revealed several networks and gene ontology terms related to the positive regulation of cellular proliferation in CK14-positive tumors. Consistent with these results, we demonstrated that the mean Ki-67 proliferative index was higher in CK14-positive tumors than it was in CK14-negative tumors (2.3 vs. 0.8%, respectively, *p* = 0.002).

**Conclusion:** CK14-positive papillary NMIUTUC is an aggressive subtype with BASQ-like molecular characteristics and dynamic proliferative activity. We propose that CK14 IHC staining can be a useful biomarker of BASQ-type papillary NMIUTUC that can be applied in daily practice with the aim of precision oncology.

## Introduction

Muscle-invasive bladder carcinoma (MIBC) is broadly classified into basal and luminal subtypes based on transcription profiles (1–5). The basal subtype shows basal/squamous/stem cell-like features: high expression of basal-type genes (*KRT5, KRT6, KRT14, CD44, CDH3, TGM1, DSC3, MYC*, and *EGFR*), low levels of luminal-type genes (*KRT20, PPARG, GATA3, FOXA1, ERBB2*, and *UPK*s), active signaling pathways [p63, c-myc, signal transducer and activator of transcription 3, and nuclear factor-κB (NF-κB)], and frequent squamous differentiation (1, 4, 6). In addition, basal-type MIBC has a worse prognosis than the luminal subtype, but it may respond well to cytotoxic chemotherapies (1, 7). Recently, it was also suggested that genetic subtypes of MIBC may determine susceptibility to immune checkpoint inhibitors (8, 9). However, the basal subtypes defined by various independent studies contain a diversity in detailed molecular and phenotypic characteristics. In an attempt to explore the common ground, therefore, a consensus was reached regarding the existence and the definition of the common basal/squamous-like (BASQ) subtype: MIBC with high expression of CK14 and CK5/6 but low expression of GATA3 and FOXA1 (10). Immunohistochemical (IHC) staining for these markers is a readily accessible technique that helps to standardize the assessment of the BASQ subtype (11, 12).

On the other hand, accumulating evidence demonstrates that non-muscle-invasive urothelial carcinoma has a clinical-pathological-genetic association that is distinct from (and occasionally even contradictory to) what has been characterized in MIBC. For example, Hedegaard and colleagues (13) demonstrated that the luminal-like (e.g., high *KRT20*/*UPK*s and low *KRT5*/*CD44*) gene expression cluster of non-muscle-invasive bladder carcinoma (NMIBC) had worse clinical outcomes than what was observed in the basal-like cluster. Different studies further support the association between luminal-like type and either advanced pathological states or short survival durations in NMIBC using CK5/6 (*KRT5/KRT6)* and CK20 (*KRT20*) as surrogate protein or mRNA markers (14–16). In line with these results when studying the urinary bladder, we previously reported that papillary non-muscle-invasive upper tract urothelial carcinoma (NMIUTUC) with luminal-like, CK5/6-negative/CK20-positive, or CD44-negative/CK20-positive, immunophenotypes had distinctly poor prognoses that were probably associated with altered cell adhesion and late cell cycle/proliferation functions (17, 18).

Despite these conflicting results observed regarding some of the subtype-defining markers in early urothelial carcinoma, high *KRT14* levels were independently prognostic of poor survival both in NMIBC and in MIBC (19). CK14 is a type I acidic keratin that is expressed in mitotically active basal cells of the stratified epithelium, where it promotes proliferation, and differentiation and in turn supports structural integrity (20). As CK14-positive basal cells differentiate into umbrella cells in the normal urothelium, CK14 expression is reduced and replaced by CK20. Consistent with this observation, Volkmer et al. (19) and Ho et al. (21) demonstrated that CK14 defined the most primitive/least differentiated basal-type urothelial carcinoma, which preceded the emergence of cancer cells expressing CK5 (intermediately differentiated) or CK20 (well-differentiated); further, CK14 expression marked the highly tumorigenic stem cell population. The increase in CK14 immunoreactivity was also observed at an early carcinogenesis stage, initiating the appearance of malignant lesions of the urinary bladder in a rat model (22). Moreover, CK14-positive urothelial carcinoma cells might have a predilection for chemoresistance (23). In addition, CK14 was associated with a poor prognosis of other malignancies, including breast cancer, squamous cell carcinoma, and salivary gland carcinoma, because it triggers proliferation, dedifferentiation, invasion, and metastasis of these cancers (24–26). However, how CK14 overexpression regulates the progression of urothelial carcinoma has not been fully elucidated. Furthermore, because of the rarity of upper tract urothelial carcinoma, which accounts for only 5–10% of the total urothelial carcinoma (27), CK14 expression and its clinicopathological and molecular significance in papillary NMIUTUC has not been studied. Herein, we investigated the prognostic implications of IHC staining for CK14 in 204 papillary NMIUTUC specimens. In addition, we analyzed the transcriptional characteristics associated with CK14 expression in papillary NMIUTUC.

## Materials and Methods

The clinical and prognostic significance of CK14 expression was determined in a prognosis cohort using IHC staining of tissue microarray (TMA) slides. Transcriptional characteristics associated with CK14 expression were evaluated in the high-grade GEP cohort composed of fresh-frozen NMIUTUC samples. As supplement, we subsidiarily assembled the low-grade NMIBC cohort using Lund urinary bladder data. This study was approved by the regional Institutional Review Board (H-1911-029-1077), and informed consent was waived by the Review Board.

### Prognosis Cohort and TMA

Formalin-fixed paraffin-embedded blocks of 204 NMIUTUC specimens (195 radical nephroureterectomy and 9 ureterectomy) were collected from the pathologic archive of Seoul National University Hospital (prognosis cohort). Clinicopathological and follow-up data were obtained from medical records. The staging and grading evaluation followed the American Joint Committee on Cancer 8th TNM staging system and World Health Organization (WHO) 2004/2016 classification, respectively (28). Triplicate 2 mm cores of tumor area were utilized for TMA experiments (Superbiochips Laboratories, Seoul, Republic of Korea).

### Immunohistochemical Staining

IHC staining for CK14 (1:300; LL002; Cell Marque Cat# 314M-14, RRID:AB_1159418, Rocklin, CA, US) and Ki-67 (1:100; MIB-1; Agilent Cat# M7240, RRID:AB_2142367, Santa Clara, CA, US) was conducted on 4-μm-thick TMA sections with a Benchmark autostainer (Ventana, Tucson, AZ, US), according to the manufacturer's instructions. IHC staining revealed that CK14 expression was variable and included the following patterns: absent, focal staining in a few tumor cells, immunoreactivity confined to the basal layer, or diffuse staining with or without occasional strong signal detected in the basal layer (Supplementary Figure 1). Because CK14 and Ki-67 stains are routinely conducted for diagnostic purposes in various organs and the staining protocols are firmly established, we did not use positive or negative controls. Considering the prognostic significance and sample distribution, immunoreactivity in >0% of tumor cells was defined as positive CK14 IHC staining (Supplementary Figure 2). Two pathologists (MJ and KCM) read the CK14 IHC slides. Intra/inter-observer variability was minimal due to the clear and straightforward staining result. The mean Ki-67 proliferative index (%) was quantitatively measured using QuPath (version 0.1.2) (29) on TMA slides that were virtually scanned (Aperio AT2, Leica Biosystem, Wetzlar, Germany). For 189 patients, IHC profiles for CK5/6 and CK20 were retrieved from our previous study (17). Briefly, positive CK5/6 and CK20 IHC staining was defined as >20 and >1%, respectively. CK5/6-other staining referred to either a positive expression or CK5/6 expression confined to the basal layer. CK20-other staining referred to either a negative reaction or CK20 expression that was restricted to umbrella cells.

### Gene Expression Profile (GEP) Cohorts

GEP analysis in the context of CK14 protein expression was determined from RNA sequencing and CK14 IHC staining data from independent high-grade papillary NMIUTUC specimens, which were generated previously (18). In short, surgically resected fresh tumors were cut in half: one half was subjected to RNA sequencing using a HiSeq 2500 platform (Illumina, San Diego, CA, US), and the other half was embedded in paraffin blocks for evaluation of CK14 immunoexpression. According to the same cutoff criteria used for CK14 positivity (>0%), 4 CK14-positive and 4 CK14-negative high-grade papillary NMIUTUC samples were assembled as the high-grade GEP cohort.

In our RNA sequencing depository, there were too few CK14-positive low-grade papillary NMIUTUC specimens to analyze. Therefore, we used Lund urinary bladder carcinoma mRNA data (GSE32894) to determine GEP related to CK14 expression in papillary low-grade early urothelial carcinoma (5). Clinicopathological details and CK14 IHC expression of these samples were obtained from another source, as summarized in Supplementary Table 1 (30). The previous annotation “urothelial-like histology,” indicating clear separation between tumor and stroma and well-arranged tumor cells with homogenous nuclei, was tentatively accepted as a papillary architecture in NMIBC (30). Consequently, a low-grade NMIBC cohort (6 CK14-positive and 25 CK14-negative NMIBCs) was created with the following inclusion criteria: (1) stage Ta or T1, (2) grade 1 [WHO 1999 guideline (28)], and (3) “urothelial-like histology.” CK14 positivity (IHC tumor cell score >0) and negativity (IHC tumor cell score = 0) were designated as above (30). For these 31 cases, gene expression data were preprocessed according to previous methods (5). Differentially expressed genes (DEG)s between CK14-positive and CK14-negative tumors were analyzed in both the high-grade GEP cohort and low-grade NMIBC cohort.

### Functional Enrichment and Network Analysis

Gene set enrichment analysis (GSEA) was performed against public gene sets (31). The networks and functional analyses were generated through the use of Ingenuity Pathway Analysis with a Benjamini-Hochberg false discovery rate (FDR) <0.05 as the cutoff (32). Based on the knowledge database, IPA determines local networks and functional terms that are particularly enriched for the input DEG sets. Gene ontology (GO) (33, 34) and Kyoto encyclopedia of genes and genomes (KEGG) (35) analyses were also performed. In addition, a Metascape membership search of GO themes was conducted, as previously described (36).

### Statistics

The association between clinicopathological variables and IHC results was analyzed by Pearson's χ*2*-test with Yate's correction or Fisher's exact test. Progression-free survival (PFS) was defined as the interval between surgery and upper urinary tract recurrence or distant metastasis, or the last follow-up visit. Cancer-specific survival (CSS) was defined as the interval between surgery and cancer-related death or the last follow-up visit. Overall survival (OS) was calculated as the term between surgery and death or the last follow-up visit. Kaplan-Meier analyses with log-rank tests were used to compare survival. A Cox proportional hazards regression model was used to calculate the hazard ratio (HR) and confidence interval (CI) in univariate and multivariate analyses. A two-tailed *p* < 0.05 was considered statistically significant. All statistical analyses were performed with SPSS 25 (IBM, Armonk, NY) or R.

## Results

### Clinicopathological Association of CK14 IHC Staining

Clinicopathological details of patients in the prognosis cohort and their association with CK14 expression are summarized in Table 1. Briefly, the median age was 67 years (range, 29–95) at diagnosis, and the male-to-female sex ratio was 4.1:1. IHC staining for CK14 was positive in 139 (68.1%) patients and negative in 65 (31.9%) patients. CK14 positivity was associated with high (≥I) TNM stage (*p* < 0.001) and high WHO grade (*p* = 0.003). In addition, CK14 positivity was related to combined CK5/6-CK20 expression (*p* = 0.001), where CK5/6-negative/CK20-positive expression was more prevalent in CK14-positive tumors (45.8 vs. 23.8%).

**Table 1:** Clinicopathological details of the prognosis cohort associated with CK14 expression.

**Variables**	**CK14-negative (*n* = 139, %)**	**CK14-positive (*n* = 65, %)**	**Total (*N* = 204, %)**	***P***
Age				0.190
<67	73 (52.5)	27 (41.5)	100 (49.0)	
≥67	66 (47.5)	38 (58.5)	104 (51.0)	
Sex				0.775
Female	26 (18.7)	14 (21.5)	40 (19.6)	
Male	113 (81.3)	51 (78.5)	164 (80.4)	
Multifocality				0.685
Absent	128 (92.1)	58 (89.2)	186 (91.2)	
Present	11 (7.9)	7 (10.8)	18 (8.8)	
TNM stage				<0.001
0	61 (43.9)	9 (13.8)	70 (34.3)	
≥I	78 (56.1)	56 (86.2)	134 (65.7)	
WHO grade				0.003
Low	86 (61.9)	25 (38.5)	111 (54.4)	
High	53 (38.1)	40 (61.5)	93 (45.6)	
CK5/6-CK20^*^				0.001
Neg-Other	22 (16.9)	12 (20.3)	34 (18.0)	
Neg-Pos	31 (23.8)	27 (45.8)	58 (30.7)	
Other-Other	34 (26.2)	3 (5.1)	37 (19.6)	
Other-Pos	43 (33.1)	17 (28.8)	60 (31.7)	

* *Data are available in 189 patients*.

*CK, cytokeratin; IVR, intravesical recurrence; TNM, tumor-node-metastasis; WHO, World Health Organization; Neg, negative; Pos, positive*.

### Prognostic Implications of CK14 IHC Staining

**Figure 1:**
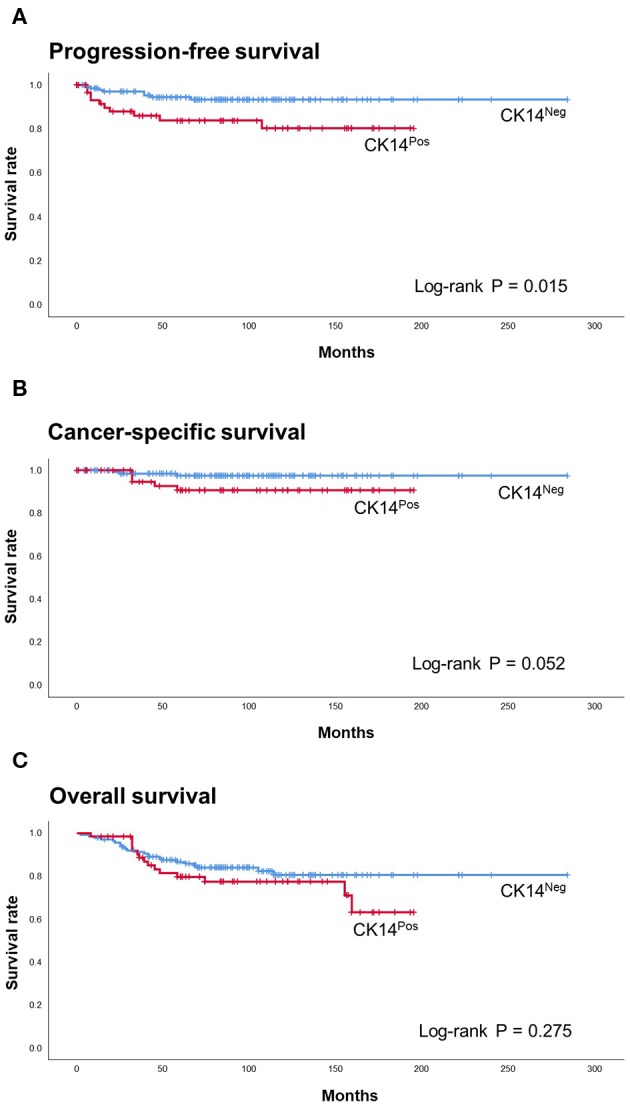
Kaplan-Meier with log-rank test of CK14 IHC staining in PFS **(A)**, CSS **(B)**, and OS **(C)** of the prognosis cohort.

In the survival analysis, CK14 positivity was significantly associated with poor PFS (*p* = 0.015; HR = 2.990; 95% CI = 1.180–7.580) and was marginally associated with poor CSS (*p* = 0.052; HR = 3.77; 95% CI = 0.900–15.780) and OS (*p* = 0.275; HR = 1.44; 95% CI = 0.750–2.760) (Figure 1). However, when adjusted for TNM stage, WHO grade and multifocality, CK14 expression failed to show significant association with PFS, while WHO grade and multifocality were independent prognostic factors of papillary NMIUTUC (Table 2).

**Table 2:** Multivariate analysis of progression-free survival.

	**Adjusted HR**	**95% CI**	***P***
CK14			
Positive vs. negative	1.995	0.731–5.448	0.178
TNM stage			
≥I vs. 0	1.308	0.442–3.867	0.628
WHO grade			
High vs. low	6.300	1.742–22.784	0.005
Multifocality			
Present vs. absent	3.515	1.130–10.934	0.030

*HR, hazard ratio; CI, confidence interval; CK, cytokeratin*.

### Transcriptional Characteristics Associated With CK14 Expression

**Figure 2:**
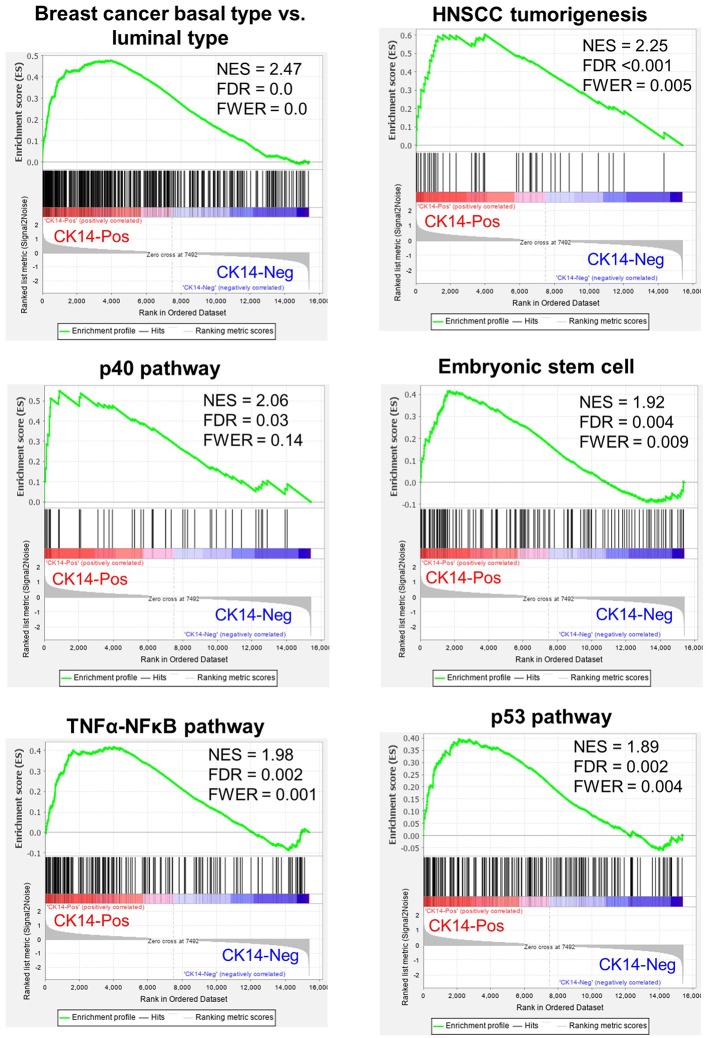
Representative GSEA results enriched in CK14-positive tumors in the high-grade GEP cohort. NES, normalized enrichment score; FDR, false discovery rate; FWER, family-wise error rate; HNSCC, head and neck squamous cell carcinoma.

The median age of the high-grade GEP cohort was 67 years (range, 56–78) with a male-to-female sex ratio of 1.7:1. The renal pelvis/calyx (50%) and ureter (50%) were equally affected. One tumor was in stage pTa, and the other 7 tumors were in pT1. After exclusion of genes with 0 fragments in any sample, 15,395 genes were identified across the high-grade GEP cohort (Supplementary Data 1). GSEA demonstrated that CK14-positive tumors were associated with a basal subtype of breast cancer (FDR = 0.0), tumorigenesis of squamous cell carcinoma (FDR <0.001), the p40 (ΔNp63) pathway (FDR = 0.03), embryonic stem cell activities (FDR = 0.004), the tumor necrosis factor-α-NF-κB pathway (FDR = 0.002) and the p53 pathway (FDR = 0.002) (Figure 2). In addition, a poorly differentiated state of squamous cell carcinoma (FDR = 0.0), c-myc (FDR = 0.002), EGFR (FDR = 0.004), Wnt (FDR = 0.004), and mammalian target of rapamycin complex 1 (mTORC1) (FDR = 0.025) cascades, cell migration (FDR <0.001) and epithelial-mesenchymal transition (EMT) (FDR = 0.025) were also significantly associated with CK14 expression in high-grade papillary NMIUTUC.

**Figure 3:**
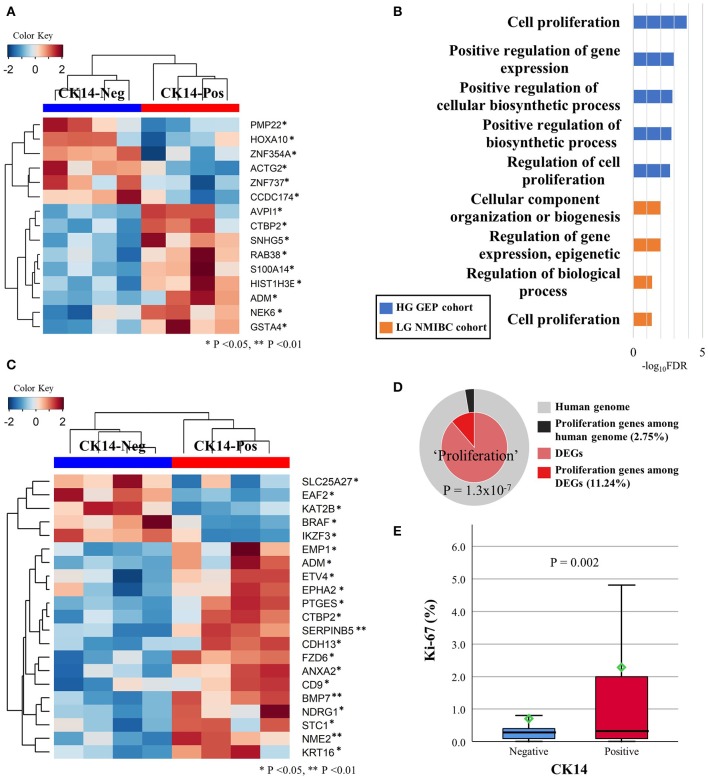
DEGs in the high-grade GEP cohort that are involved in cellular growth and proliferation network, as revealed by IPA **(A)**. Several GO-biologic process terms associated with proliferation were identified in the high-grade GEP cohort (blue) and the low-grade NMIBC cohort (orange) **(B)**. Unsupervised clustering analysis of the DEGs included in the “cell proliferation” (GO: 0008283) GO term in the high-grade GEP cohort **(C)**. Membership search of proliferation-related GO signatures enriched in the high-grade GEP cohort **(D)**. The Ki-67 proliferative index is much higher in CK14-positive tumors than it is in CK14-negative tumors in the prognosis cohort **(E)**.

With a *p* < 0.05 and |fold change| ≥2 as the cutoffs, we identified 178 DEGs in the high-grade GEP cohort; of those identified, 103 and 75 genes were highly and lowly expressed, respectively, in CK14-positive tumors (Supplementary Figure 3A). *KRT14* gene was highly expressed in CK14-positive tumors compared to CK14-negative tumors (fold change = 40.2, *p* = 0.035). IPA identified “cellular growth and proliferation” as one of the highest scored networks specifically related to CK14 expression (Figure 3A, Supplementary Figure 4). Other network-associated functions that were influenced by CK14 expression mainly included “cellular movement” and “cancer” (Table 3). IPA also identified significant enrichment of cellular movement/invasion and cell death functions, which were upregulated and downregulated, respectively, in CK14-positive tumors (Table 4). Consistent with these results, several GO and KEGG terms related to cell proliferation and cell component biosynthesis (e.g., “cell proliferation,” FDR <0.001; “positive regulation of gene expression,” FDR = 0.001), cell death (e.g., “cell death” and “apoptotic process”; both, FDR <0.001), adhesion (e.g., “cell adhesion” and “biologic adhesion”; both, FDR <0.001) and “pathways in cancer” (FDR <0.001) were identified (Figures 3B,C, Supplementary Table 2). Furthermore, GO themes related to proliferation (“cell proliferation,” “positive regulation of cell proliferation,” “epithelial cell proliferation,” and “regulation of epithelial cell proliferation”) were particularly enriched within the DEGs when compared to their representation within the human genome (*p* = 1.3 × 10^−7^) (Figure 3D).

**Table 3:** Networks associated with CK14 positivity as assessed by Ingenuity Pathway Analysis.

**Network**	**DEGs**	**Score**
*High-grade GEP cohort*		
Digestive system development and function, gastrointestinal disease, organ morphology	*NIPAL2, AIFM3, EMP1, PLK2, TBC1D3, CAPN2, ARHGAP32, NPIPA8, BRAF, MPZL2, LMO7, SERPINB5, PERP, COL17A1, KRT14, ERCC5*	22
Cellular growth and proliferation, cellular development, gene expression	*PMP22, HOXA10, ZNF354A, ACTG2, ZNF737, CCDC174, AVPI1, CTBP2, SNHG5, RAB38, S100A14, HIST1H3E, ADM, NEK6, GSTA4*	20
Cellular movement, cancer, organismal injury and abnormalities	*SPINK5, SALL2, ANKS1A, ATP2B4, SERPINB5, EPHA2, ACSM3, PON3, ANO1, CLDND1, AHNAK, TAGLN, ACTA2, PSIP1, EPOR*	20
Cellular movement, cellular development, tissue development	*ITGA6, ALDH161, CD9, PTGFRN, CHAF1A, PTGES, POU5F1, KAT2B, BAG3, BRAF, KRT16, CD56, NDUFC2, RPL10A, RND3*	20
Cellular growth and proliferation, connective tissue development and function, tissue development	*KCNK1, RELB, MPZL2, MAP3K8, USP6NL, RAD54B, SLC2A1, PERP, NDRG1, GPR87, PLK2, ETHE1, ATIC, EAF2*	18
*Low-grade NMIBC cohort*		
Cellular growth and proliferation, cell cycle, cellular development	*IGF2, IL20RB, COL7A1, HIST1H2AC, HIST1H2BG, HIST2H2BE, HIST2H2AA3, HIST2H2AA4, TRNP1, IL1A, RND3*	24

*DEGs, differentially expressed genes; NMIUTUC, non-muscle-invasive upper tract urothelial carcinoma; NMIBC, non-muscle-invasive bladder carcinoma*.

**Table 4:** Functional enrichment in CK14-positive tumors in the high-grade GEP cohort as assessed by Ingenuity Pathway Analysis.

**Category**	**Function**	Activation z-score^*^	**FDR**
Cellular Movement	Invasion of tumor cell lines	1.491	0.00251
	Cell movement of tumor cell lines	1.947	0.00255
	Invasion of cells	1.674	0.00255
	Migration of tumor cell lines	2.185	0.0058
	Migration of cells	1.975	0.0169
	Cell movement	1.658	0.0182
Cell death and survival	Necrosis	−1.246	0.00218
	Apoptosis	−1.159	0.00316
	Cell death of tumor cell lines	−1.361	0.00393
	Apoptosis of tumor cell lines	−1.658	0.0274

*GEP, gene expression profile; FDR, false discovery rate (Benjamini Hochberg)*.

* *z-scores indicate activation (>0) or inhibition (<0) of the predicted function based on the fold change of the DEGs (upregulation or downregulation) and its agreement with the functions*.

A similar comparison of the low-grade NMIBC cohort identified 26 DEGs between CK14-positive and CK14-negative tumors. In unsupervised clustering analysis, all CK14-positive tumors and 3 CK14-negative tumors (UC_0041_2, UC_0240_1 and UC_0414_1) were grouped together by these DEGs (Supplementary Figure 3B). There was one gene (*CLCA4*) that was upregulated in CK14-positive tumors both in the high-grade GEP cohort and the low-grade NMIBC cohort. In agreement with the high-grade GEP cohort, cellular growth and proliferation overrepresented the biological functions associated with CK14 expression in the low-grade NMIBC cohort, as revealed by the IPA network search (Table 3, Supplementary Figure 5) and GO analysis (Figure 3B, Supplementary Table 2).

Next, we tried to validate the difference in cellular proliferation between CK14-positive and CK14-negative papillary NMIUTUC using Ki-67 IHC staining. CK14-positive tumors displayed a 2.3 ± 5.36% proliferative index (mean ± standard deviation), which was higher than 0.8 ± 2.06% index of CK14-negative tumors in the prognosis cohort (Mann-Whitney U, *p* = 0.002) (Figure 3E).

## Discussion

In this study, we demonstrated that positive IHC staining for CK14 was associated with high-risk phenotypes of papillary NMIUTUC and was predictive for unfavorable survival rates. We compared CK14-positive and CK14-negative high-grade papillary NMIUTUC at the mRNA level to define a molecular framework underlying CK14 expression in papillary NMIUTUC. We found that CK14 positivity in papillary high-grade NMIUTUC marked transcriptional characteristics reminiscent of those in BASQ-type MIBC, including signatures of basal/stem cell and squamous cell carcinoma. This CK14-positive BASQ-like subtype was characterized by high proliferative functions in high-grade papillary NMIUTUC as well as in low-grade papillary NMIBC. IHC staining for Ki-67 verified a much higher proliferative activity in CK14-positive tumors than what was seen in CK14-negative papillary NMIUTUC.

There have been several seminal studies that uncovered genetically heterogeneous subclasses within MIBC via transcriptional clustering (1–5, 37). Of the subclasses, a common subtype, named the BASQ subtype, harbors a high level of basal-type genes, squamous-like features, and an aggressive phenotype (10). Although the BASQ subtype is a good foundation for MIBC evaluation, the applicability of BASQ markers (CK14, CK5/6, GATA3, FOXA1) to NMIUTUC or NMIBC has been questioned by us and others (13, 14, 16–18). For instance, CK5/6-negative/CK20-positive luminal-like papillary NMIUTUC had worse survival and was enriched with motility- and proliferation-associated genes (17, 18). In the same way, “class 2” NMIBC defined by Hedegaard and coworkers (13) was characterized by luminal-like genetic attributes, including low *KRT*5, *KRT6*, and *CD44* expression and high *GATA3, FOXA1*, and *KRT20* expression, but it had far worse survival than the other types of NMIBC. Moreover, this “class 2” subtype highly expressed *KRT14* and genetic signatures related to late cell cycle, cancer stem cell phenotype and EMT, which was in accord with another report that found high CK14/*KRT14* to be a determinant of poor NMIBC prognosis (19). In contrast to “class 2,” basal-like subtype “class 3” NMIBC was paradoxically shown to have a favorable prognosis, which probably reflected a dormant state (13). Consistent with these reports, we showed that CK14 positivity was associated with poor PFS in papillary NMIUTUC, which was in agreement with the characteristics of CK14-positive BASQ-type MIBC. Although the overall good prognosis of papillary NMIUTUC hindered the statistical significance of CK14 IHC staining in predicting CSS and OS in our study, Kaplan-Meier plots suggested that CK14 positivity also reached worse outcomes in CSS and OS. In addition, we demonstrated that CK14-positive papillary NMIUTUC had BASQ-like genetic features that were similar to those of basal-type breast cancer and those of squamous cell carcinoma (1, 2, 4, 38). Furthermore, CK14-positive papillary NMIUTUC shared other molecular hallmarks of BASQ-type MIBC: activated p40, c-myc, EGFR, and NF-κB pathways, which mediate growth and squamous differentiation of urothelial carcinoma. These observations further support the genetic similarity between CK14-positive papillary NMIUTUC and BASQ-type MIBC (1, 6, 38).

Network and functional enrichment analyses as well as Ki-67 IHC staining demonstrated that cellular growth and proliferative functions are particularly upregulated in CK14-positive papillary NMIUTUC, in accordance with previously reported high-risk phenotypes of NMIBC (13) and NMIUTUC (18). The DEGs associated with this function involved several oncogenes [e.g., *CTBP2* (39), *SNHG5* (40), *RAB38* (41), *ADM* (42), *ETV4* (43), *EPHA2* (44), *PTGES* (45), *FZD6* (46), *ANXA2* (47), and *STC1* (48)] and tumor-suppressor genes [e.g., *EAF2* (49) and *IKZF3* (50)], which were upregulated and downregulated, respectively, in CK14-positive tumors. Dysregulation of these genes modulates proliferation, survival, migration, EMT, or metastasis of urothelial carcinoma, and other malignancies, leading to poor prognoses of these tumors. Interestingly, some of these genes, such as *ETV4, PTGES, CTBP2, FZD6, EAF2*, and *IKZF6*, have not been implicated in urothelial carcinoma; thus, these genes are potential candidates for diagnostic, prognostic, and therapeutic biomarkers of urothelial carcinoma. In addition to cellular proliferation, functional analyses suggested that CK14-positive papillary NMIUTUC had increased invasion and migration activities. In early urothelial neoplasms, CK14 expression denotes a stem cell population of basal cells that harbor high mitotic activities and have self-renewal, tumorigenesis, and stromal invasion potential (21, 51). Likewise, varied transcription activators and activation pathways (e.g., p40, c-myc, NF-κB, EGFR, Wnt, and mTORC1 signaling pathways) that were enriched in CK14-positive papillary NMIUTUC are linked to proliferation and/or malignant transformation of basal cells and embryonal stem cells (21). For example, silencing of p40 inhibited c-myc-mediated proliferation of urothelial carcinoma, and high p63 expression was associated with poor prognosis in NMIBC (6, 21). Both EGFR-dependent cell growth and EGFR inhibitor-induced growth restriction were specific to the BASQ subtype in MIBC (38). CK14 expression may regulate the treatment response to cancer therapeutics, including chemotherapy, immunotherapy, and Bacillus Calmette-Guérin in urothelial carcinoma (1, 7–10, 19, 21, 52). Therefore, we hypothesize that CK14-positive papillary NMIUTUC is highly populated with proliferative cancer stem cells, which will potentially impact the treatment strategy.

Normal urothelium has a hierarchical structure of basal, intermediate, and umbrella cells with sequential differentiation that involve gradual changes in keratin profiles from CK14 to CK5 and/or to CK20; thus, these cells that are in different states supposedly can give rise to urothelial carcinomas with poor (CK14-positive/CK5-negative/CK20-negative), moderate (CK14-negative/CK5-positive/CK20-negative), and well (CK14-negative/CK5-negative/CK20-positive) differentiation, respectively (19, 21). The BASQ-like molecular subtypes defined in different studies, including the TCGA cluster III/IV, the Lund “urothelial-like B” subtype and the Lund “squamous cell carcinoma-like” subtype, exhibited poorly differentiated gene expression characteristics (10). Interestingly, poorly differentiated properties associated with CK14 positivity might be partially opposed by our finding that CK14 positivity was associated with luminal-like CK5/6-negative/CK20-positive expression in papillary NMIUTUC (17). The Lund “genomically unstable” subtype, which corresponds to a high-risk group of NMIBC, expresses low CK5 and high CK20, and is enriched with genetic signatures typical of urothelial differentiation and disrupted adhesion (5, 10, 14, 30). Similarly, low CK5/6 and high CK20 expression profiles were associated with high-risk phenotypes of both papillary NMIUTUC and NMIBC that exhibited altered adhesion, migration, and proliferation (13, 17, 18). Both CK14 and CK20 were suggested to direct the aggravation of early urothelial carcinoma via the carcinoma *in situ*-driven pathway (13, 17, 21). In an animal model, altered expression of CK14 and CK20 corresponded to an early tumorigenic step of papillary urothelial carcinoma (22). Taken together, it would be reasonable to speculate that CK5/6-negative/CK20-positive expression may represent a “second hit” to a primitive and aggressive CK14-positive papillary NMIUTUC. In other words, this putative CK14-positive/CK5/6-negative/CK20-positive expression might mirror a highly progressive CK14-positive urothelial carcinoma progeny, which remains to be explored (19). Furthermore, CK14, CK5/6, and CK20 expression would be valuable prognostic biomarkers in papillary NMIUTUC that appear to complement one another.

This study has some limitations. The number of patients included in the prognosis cohort was not enough to statistically discriminate CSS and OS in survival analysis. Second, the high-grade GEP cohort was relatively small, which introduced a risk of a biased estimation regarding molecular characteristics. Nevertheless, transcriptional profiles of CK14-positive papillary NMIUTUC were similar to those reported for the BASQ subtype, underscoring the appropriate representation of the high-grade GEP cohort. Finally, we applied array-based NMIBC mRNA data to infer transcriptional changes influenced by CK14 IHC staining in low-grade tumors. It is known that urothelial carcinoma of the upper and lower tract has high amount of overlap in transcriptomes, especially within early-staged tumors (53). However, the differences in tumor locations and analytic platforms prohibited deeper comparison of the high-grade GEP cohort and the low-grade NMIBC cohort. Further study incorporating enough samples of both low-grade and high-grade papillary NMIUTUC that exhibits positive CK14 IHC staining will be required.

In conclusion, CK14 positivity represents an aggressive BASQ-like subtype in papillary NMIUTUC that is enriched with brisk proliferative activity. CK14 IHC staining is a promising biomarker that can be applied in the management of non-muscle-invasive urothelial carcinoma in daily practice, with the aim of precision oncology.

## Data Availability Statement

Publicly available datasets were analyzed in this study, these can be found in the NCBI Gene Expression Omnibus (GSE32894). The datasets generated for this study can be found in the Sequence Read Archive (SRA), PRJNA609154.

## Ethics Statement

The studies involving human participants were reviewed and approved by Institutional Review Board of Seoul National University Hospital. Written informed consent for participation was not required for this study in accordance with the national legislation and the institutional requirements.

## Author Contributions

MJ and KM designed and performed the experiments. IJ, KK, and MJ analyzed the data. MJ wrote the manuscript. KM supervised the entire process.

## Conflict of Interest

The authors declare that the research was conducted in the absence of any commercial or financial relationships that could be construed as a potential conflict of interest.
