# Supplementary material for: CK14 Expression Identifies a Basal/Squamous-Like Type of Papillary Non-Muscle-Invasive Upper Tract Urothelial Carcinoma
Source: Front Oncol. 2020 Apr 24;10:623. doi: 10.3389/fonc.2020.00623 (PMC7193093; doi:10.3389/fonc.2020.00623)
Supplement: Supplementary file 2 [file Data_Sheet_2.docx]

Supplementary Material

# Supplementary Figures

**
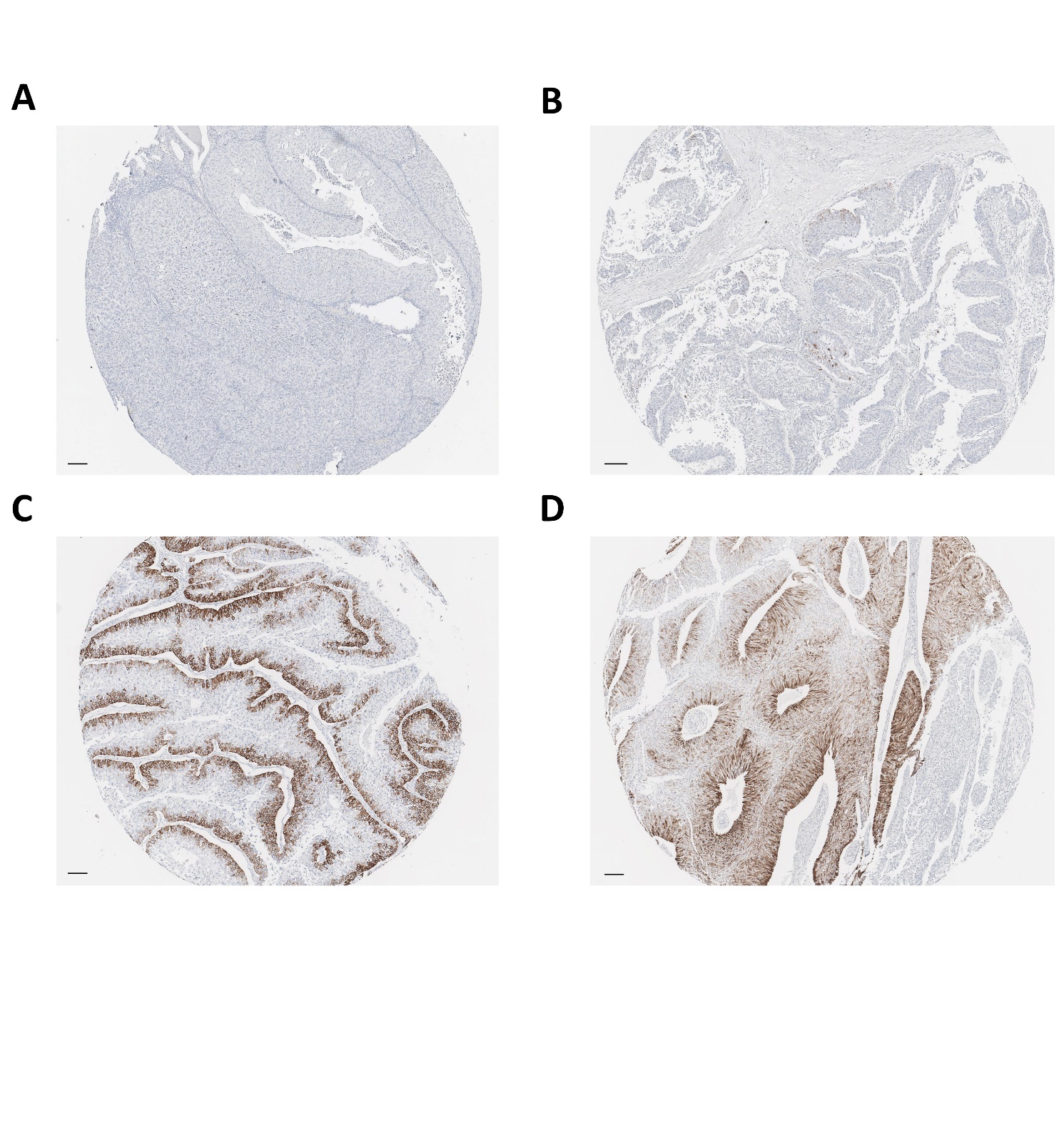
**

**Supplementary Figure 1.** CK14 IHC staining is observed variously: absent (0%) **(A)**, stained in a few tumor cells (0-1%) **(B)**, confined to the basal layer (>1%) **(C)**, and diffusely reactive with occasional accentuation in the basal layer (>1%) **(D)**. scale bar = 100 μm

**
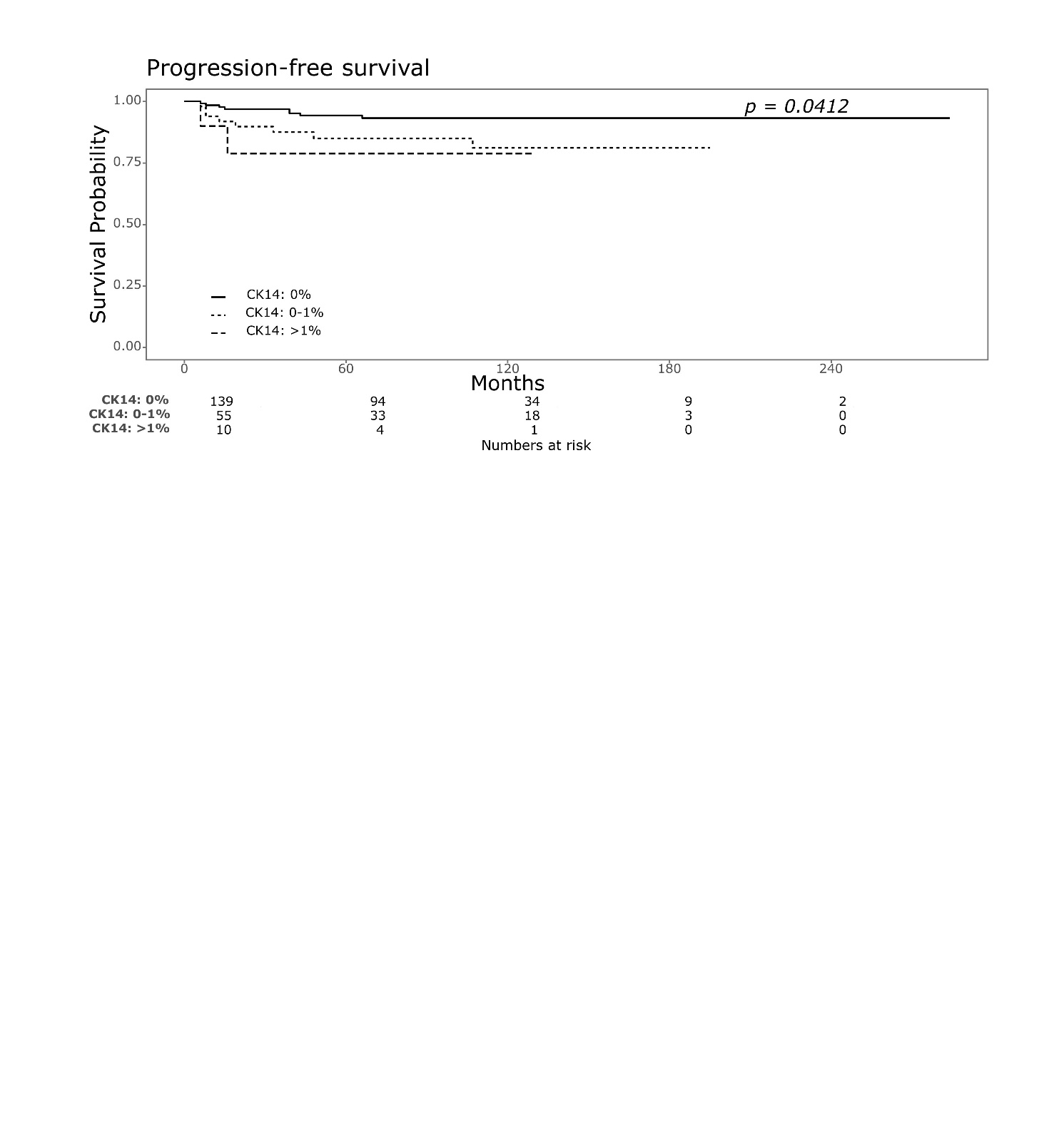
**

**Supplementary Figure 2.** A 0% group (0% CK14 staining in all TMA cores; n = 139) showed distinctly poor PFS than a >1% group (>1% staining in all TMA cores; n = 10) or the remaining specimens (0-1% staining in any TMA cores; n = 55), which were similarly observed in survival analyses for CSS and OS. Therefore, the cutoff value of CK14 positivity was determined as >0%. (Visualization: <http://web-r.org/>)


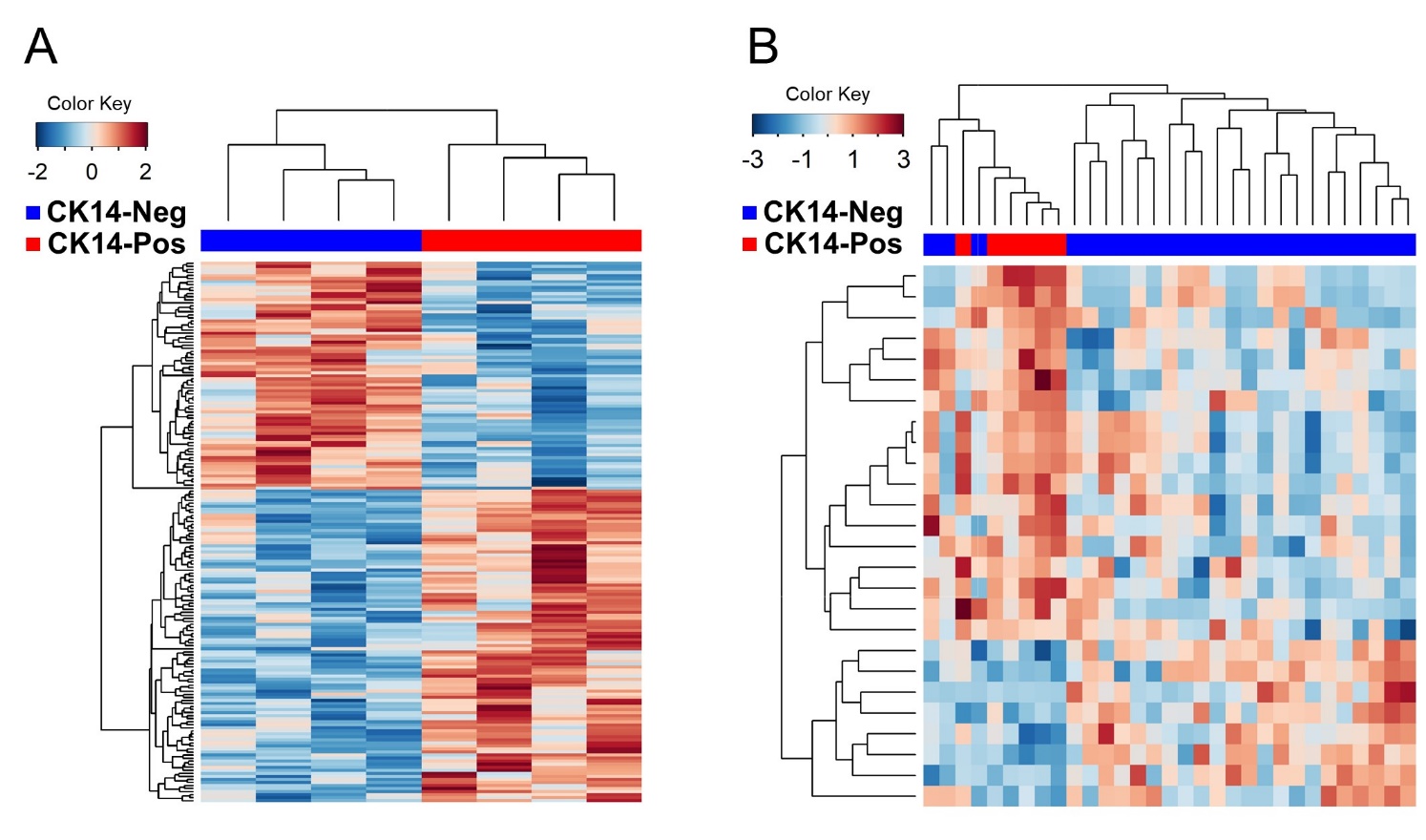


**Supplementary Figure 3.** Unsupervised clustering analysis of DEGs between CK14-positive and CK14-negative tumors in the high-grade GEP cohort and **(A)** the low-grade NMIBC cohort **(B)**.


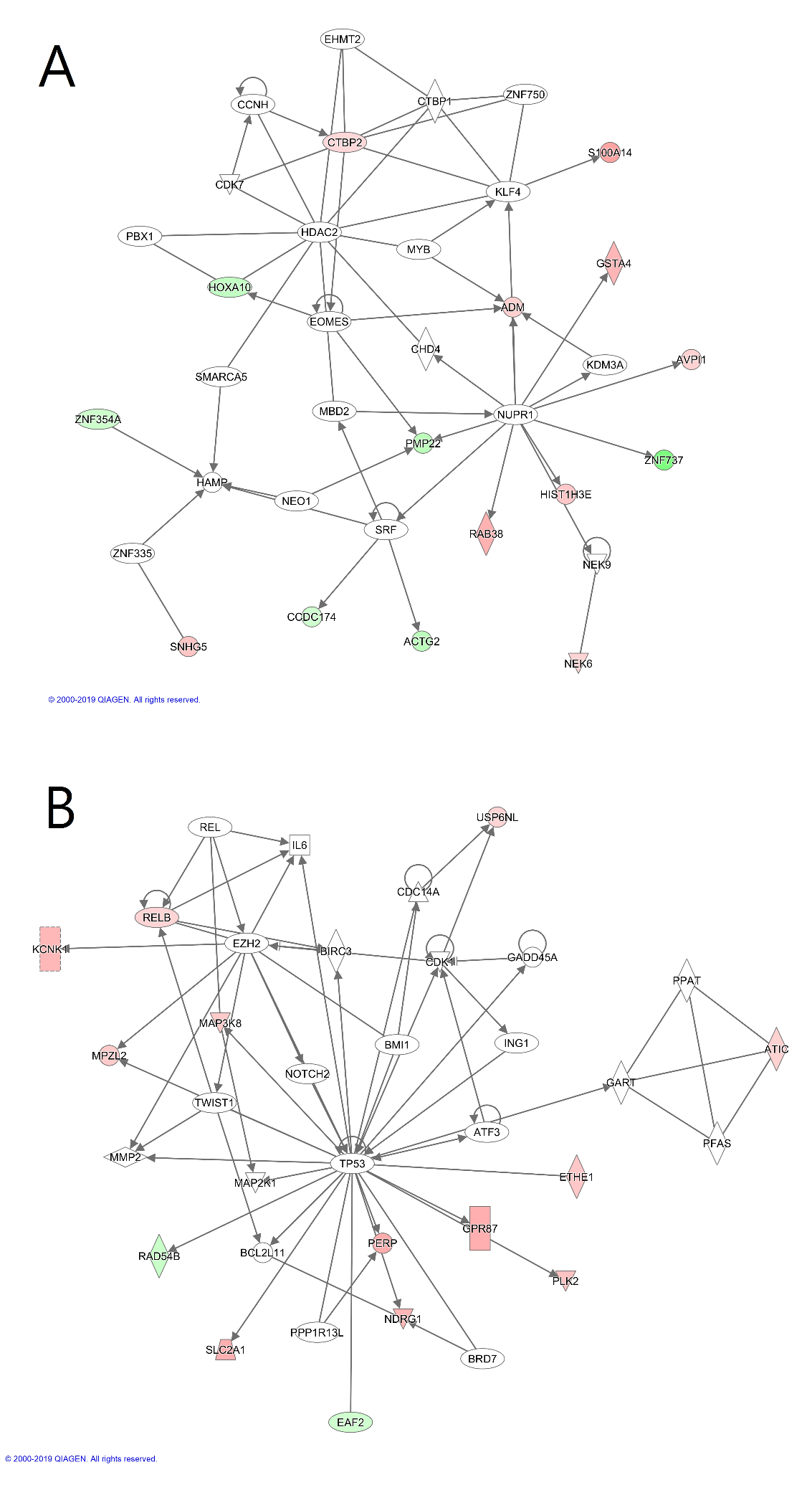


**Supplementary Figure 4.** Cellular growth and proliferation, cellular development, gene expression network **(A)** and cellular growth and proliferation, connective tissue development and function, tissue development network **(B)** are differentiating between CK14-positive and CK14-negative papillary NMIUTUC.


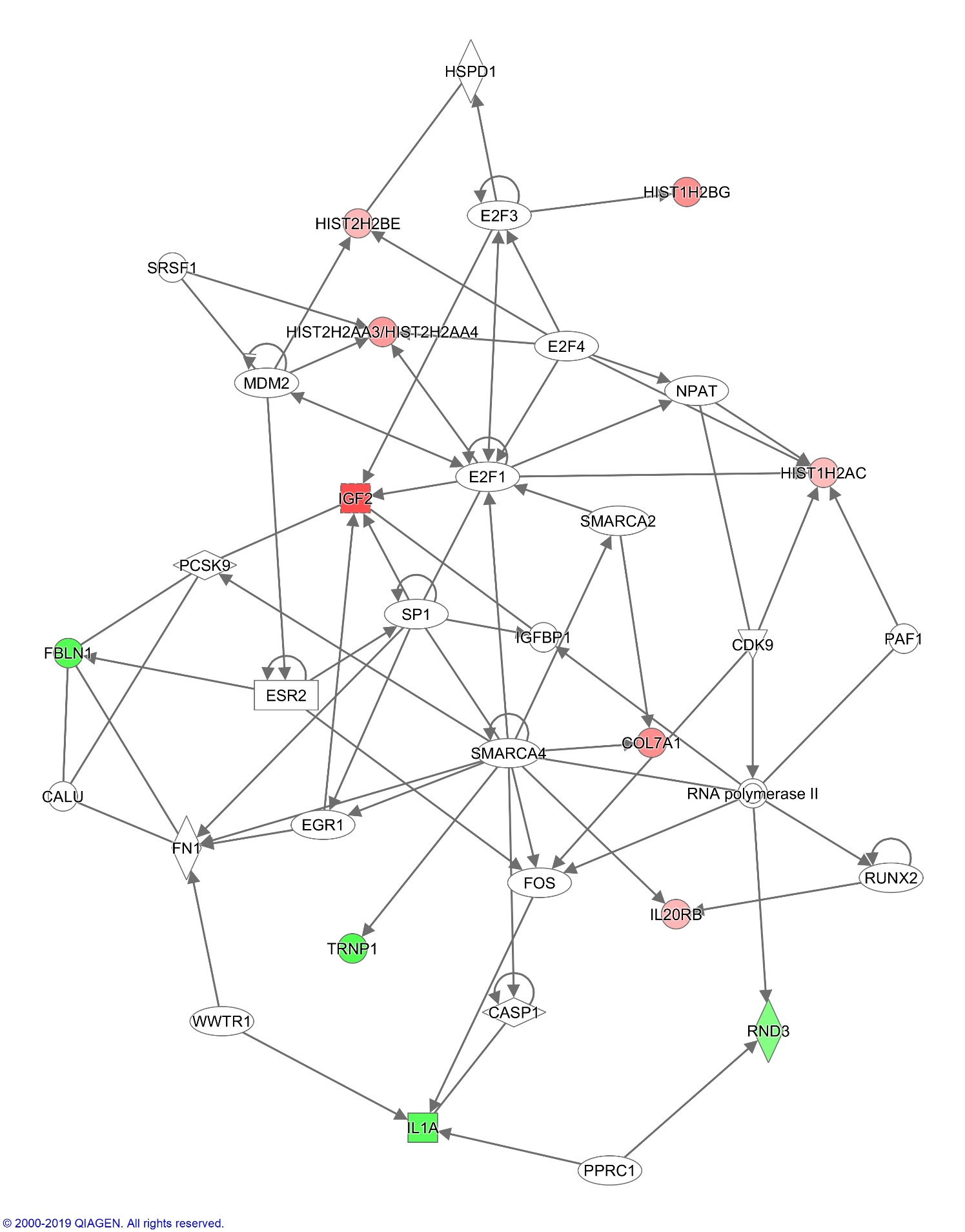


**Supplementary Figure 5.** Cellular growth and proliferation, cell cycle, cellular development is the top network affected by CK14 IHC staining in papillary NMIBC.

# Supplementary Tables

| **Supplementary Table 1. Clinicopathological characteristics and molecular subtypes of the low-grade NMIBC cohort** | | | | |
| --- | --- | --- | --- | --- |
| Variables | CK14-negative (n = 25, %) | CK14-positive (n = 6, %) | Total (N = 31, %) | P |
| Age |  |  |  | 0.587 |
| <68 | 11 (44.0%) | 4 (66.7%) | 15 (48.4%) |  |
| ≥68 | 14 (56.0%) | 2 (33.3%) | 16 (51.6%) |  |
| Sex |  |  |  | 1.000 |
| Female | 10 (40.0%) | 3 (50.0%) | 13 (41.9%) |  |
| Male | 15 (60.0%) | 3 (50.0%) | 18 (58.1%) |  |
| T stage |  |  |  | n.a. |
| 0 | 25 (100.0%) | 6 (100.0%) | 31 (100.0%) |  |
| 1 | 0 (0.0%) | 0 (0.0%) | 0 (0.0%) |  |
| WHO grade |  |  |  | n.a. |
| G1 | 25 (100.0%) | 6 (100.0%) | 31 (100.0%) |  |
| G2 or 3 | 0 (0.0%) | 0 (0.0%) | 0 (0.0%) |  |
| CK14 (median TCS) | 0.0 | 0.5 | 0.0 | <0.001 |
| Lund molecular subtype |  |  |  | 0.470 |
| MS1a | 10 (40.0%) | 4 (66.7%) | 14 (45.2%) |  |
| MS1b | 15 (60.0%) | 2 (33.3%) | 17 (54.8%) |  |

Abbreviation: WHO, World Health Organization; TCS, tumor cell score

| **Supplementary Table 2. Top 5 gene ontology (GO) and Kyoto encyclopedia of genes and genomes (KEGG) terms** | | | | |
| --- | --- | --- | --- | --- |
| Category | High-grade GEP cohort | | Low-grade NMIBC cohort | |
|  | Terms (ID) | FDR | Terms (ID) | FDR |
| GO-BP | Cellular process (GO:9987) | 1.36728x10^-33^ | Cellular component organization (GO:0016043) | 0.00962 |
|  | Biological regulation (GO:65007) | 4.71759x10^-28^ | Cellular process (GO:0009987) | 0.00962 |
|  | Single-organism process (GO:44699) | 7.54751x10^-27^ | Cellular component organization or biogenesis (GO:0071840) | 0.01095 |
|  | Regulation of biological process (GO:50789) | 5.01092x10^-24^ | Single-organism process (GO:0044699) | 0.01097 |
|  | Regulation of cellular process (GO:50794) | 5.23257x10^-23^ | Regulation of gene expression, epigenetic (GO:0040029) | 0.01109 |
| GO-CC | Cell part (GO:44464) | 1.23237x10^-36^ | Extracellular region (GO:0005576) | 0.00000 |
|  | Cell (GO:5623) | 1.23237x10^-36^ | Membrane-bounded vesicle (GO:0031988) | 0.00001 |
|  | Organelle (GO:43226) | 2.86317x10^-32^ | Vesicle (GO:0031982) | 0.00001 |
|  | Intracellular (GO:5622) | 2.55996x10^-30^ | Extracellular region part (GO:0044421) | 0.00001 |
|  | Membrane-bounded organelle (GO:43227) | 1.87623x10^-28^ | Extracellular membrane-bounded organelle (GO:0065010) | 0.00003 |
| GO-MF | Binding (GO:5488) | 5.62653x10^-31^ | Binding (GO:0005488) | 0.00004 |
|  | Protein binding (GO:5515) | 3.46704x10^-21^ | Protein heterodimerization activity (GO:0046982) | 0.00021 |
|  | Ion binding (GO:43167) | 7.58821x10^-13^ | Protein binding (GO:0005515) | 0.00395 |
|  | Heterocyclic compound binding (GO:1901363) | 3.28961x10^-10^ | Protein dimerization activity (GO:0046983) | 0.00860 |
|  | Organic cyclic compound binding (GO:97159) | 5.17409x10^-10^ | n.a. | n.a. |
| KEGG | Pathways in cancer (5200) | 0.00032 | Systemic lupus erythematosus (5322) | 0.00000 |
|  | Vascular smooth muscle contraction (4270) | 0.00032 | Alcoholism (5034) | 0.00000 |
|  | Herpes simplex virus 1 infection (5168) | 0.00123 | Necroptosis (4217) | 0.00011 |
|  | Metabolic pathways (1100) | 0.00123 | n.a. | n.a. |
|  | Human T-cell leukemia virus 1 infection (5166) | 0.01921 | n.a. | n.a. |

Abbreviation: GEP, gene expression profile; ID, identification; FDR, false discovery rate; GO, gene ontology; BP, biologic process; CC, cellular component; MF, molecular function; n.a., not available; KEGG, Kyoto encyclopedia of genes and genomes
